# Cross-sectional associations of self-reported firearm use with blood lead concentrations in a nationally representative cohort of US adults

**DOI:** 10.1097/EE9.0000000000000427

**Published:** 2025-10-23

**Authors:** Madeline Day, Joseph M. Braun, Christian Hoover

**Affiliations:** a Department of Epidemiology, Brown University, School of Public Health, Providence, Rhode Island; b Harvard University Injury Control Research Center, Harvard T.H. Chan School of Public Health, Boston, Massachusetts

## Abstract

**Background:**

Firearm-related lead exposure could occur when firearms discharge lead ammunition particles. Prior studies were primarily among military or police participants, but this relation has not been examined in civilian populations. Thus, among noninstitutionalized United States (US) adult civilians, we examined the association of self-reported firearm noise exposure (proxy for firearm-related lead exposure) and firearm use with blood lead concentrations.

**Methods:**

We used 5 cycles of the National Health and Nutrition Examination Survey. Using lead biomarkers and questionnaires related to firearm noise exposure (1999–2004, n = 9,606) and firearm use (2011–2012 and 2015–2016, n = 5,972), we calculated survey-weighted and covariate-adjusted percent differences in blood lead concentrations. We adjusted for gender, age, race/ethnicity, and education. Sensitivity analyses separately examined whether former military status, pre-1978 housing, or occupation confounded these associations.

**Results:**

Self-reported firearm noise exposure was associated with blood lead concentrations (percent difference 15%; 95% CI = 7%, 23%), but firearm use was not (percent difference 1%; 95% CI = −5%, 9%). However, blood lead concentrations were 9% (95% CI = −5%, 25%) and 21% (95% CI = −5%, 54%) higher among those who reported shooting 1,000–10,000 and 10,000+ rounds, respectively (*P* value for trend = 0.07), compared with those who reported shooting 0 rounds. Results were similar after separate adjustment for former military status, pre-1978 housing, and occupation.

**Conclusion:**

In this representative sample of US civilians, individuals who used firearms more frequently (10,000+ rounds) had increased blood lead concentrations.

**What this study adds**

Given that firearm-related lead exposure is now a common nonoccupational source of lead in the United States and that prior studies have primarily been conducted in military or police settings, our study contributes to an understanding of the phenomenon in civilian populations. Our research would be of interest to readers studying emerging sources of lead exposure and may have potential implications for US adult lead screening guidelines.

## Introduction

Lead exposure is a major health concern given its neurotoxicity.^1–4^ Lead disrupts the release of neurotransmitters, decreasing cognitive and executive functions.^4–6^ Chronic lead exposure is associated with increased aggressiveness, impulsivity, and risk of major depressive disorders, as well as decreased attention span, memory, and cognitive processing.^2,7–9^ Additional chronic effects include cardiovascular, renal, reproductive, and other diseases ^2,3,9^

Though blood lead concentrations of 50 μg/L and above in adults are considered elevated by the Centers for Disease Control and Prevention (CDC) Adult Blood Lead Epidemiology and Surveillance (ABLES) program, no levels of exposure are safe.^10^ While exposure to lead in the United States has declined since the 1970s, blood lead concentrations below 50 μg/L are still associated with intellectual impairments and deleterious health outcomes, including poor renal function and cardiovascular disease.^11,12^ For example, US adults with blood lead concentrations above 24 μg/L had a higher risk of decreased renal function compared with those with blood lead concentrations below 11 μg/L.^13^ Consistent with low-level effects in adults, Lanphear and colleagues^14^ found that among children between 5 and 10 years old, an increase in blood lead concentration from 24 to 100 μg/L was associated with an inverse 3.9 unit change in total IQ score. In 2017–2018, 5% of US adults older than 20 had blood lead concentrations at or above 26.2 μg/L.^15^ Such persistent levels of lead, despite declines in legacy-based lead exposure sources like paint and water lines, suggest the need to address additional contemporary sources of lead, such as firearms.

Lead ammunition could be responsible for firearm-related lead exposures in multiple ways.^16^ First, firearm users inhale lead particles and fumes ejected from lead bullet fragments and lead-containing primer when firing.^16^ Second, lead dust and particles settle on users’ clothing and personal items.^16^ Users then track lead particles into their home or work environments (“take home” exposures).^17^ Third, the consumption of game animals killed by lead ammunition may also expose users who hunt.^18^ Fourth, lead accumulates in hunting ground or outdoor firing range soil, serving as a source of exposure.^19,20^

Recent findings demonstrate that US household firearm ownership is positively correlated with pediatric blood lead concentrations at the city, town, and state levels.^21^ Across 44 states between 2012 and 2018, for every 14% increase in household firearm ownership, the prevalence of elevated pediatric blood lead concentrations increased by 41%.^21,22^

Shooting in indoor firing ranges is also associated with elevated blood lead concentrations.^23,24^ An Italian study comparing firearm-exposed participants (firearm instructors) and nonfirearm-exposed participants (food plant workers) found associations between firearm exposure and blood lead concentrations.^24^ A South African study comparing the blood lead concentrations of recreational firearm users and archers found that 80% of users had blood lead concentrations greater than 50 µg/L, compared to 23% of archers.^9^ Furthermore, cross-sectional studies of police officers found evidence of a dose-response relationship between firearm use and blood lead concentrations.^25^

A notable gap in the literature is the lack of understanding of firearm-related lead exposure in civilians without occupational use of firearms. Studies have primarily examined the impact of firearm use in military, police, or other occupational settings.^22,26–31^ This means that these studies have been conducted in predominantly male populations over short-term exposure periods.^22,26–31^ Contemporary literature has not examined firearm-related lead exposure at the population level, through nationally representative data, or over multi-year periods. Increased understanding of firearm-related lead exposure in civilians could inform lead screening guidelines for US adults.^22,26–31^

To address this gap, we examined whether self-reported firearm noise exposure and firearm use are associated with blood lead concentrations in noninstitutionalized US civilians aged 20–69 using the 1999–2004, 2011–2012, and 2015–2016 cycles of the National Health and Nutrition Examination Survey (NHANES).

## Methods

### Study design and setting

The NHANES is a cross-sectional, nationally representative survey of noninstitutionalized US civilians. During each 2-year survey cycle, NHANES selects approximately 10,000 participants through probability sampling. Participants take part in household interviews and physical examinations. We used five cycles of NHANES data from 1999–2000, 2001–2002, 2003–2004, 2011–2012, and 2015–2016 for these analyses. Data are publicly available and retrieved from the National Center for Health Statistics (NCHS) https://wwwn.cdc.gov/nchs/nhanes/Default.aspx.^32^ Institutional review board approval was not required because our analyses used publicly available de-identified data.

### Study participants

We selected survey cycles based on the availability of questionnaires related to firearm use or firearm noise-related exposure. Given that the wording and number of questions related to firearms differed across cycles, we conducted two sets of analyses: one for the 1999–2004 surveys and another for the 2011–2012 and 2015–2016 surveys. After selecting participants aged 20–69 with complete data (firearm questionnaire, blood lead concentrations, and relevant covariates) and excluding former military service members, 9,606 participants remained for 1999–2004 and 5,972 participants remained for 2011–2012 and 2015–2016. Former military service members were excluded to reduce the influence of military- or combat-related lead exposures.

### Firearm use

As part of the audiometry questionnaire, self-reported firearm noise exposure data were collected in 1999–2004 for participants aged 20 and above, while data on firearm use and number of rounds shot were collected in 2011–2012 and 2015–2016 for participants aged 20–69. This data was not collected in 2013–2014. For the 1999–2004 survey cycles, we restricted participant age to 20–69 for consistency with 2011–2012 and 2015–2016.

In the 1999–2004 survey cycles, we assessed self-reported firearm noise exposure using the question (Audiometry (AUQ),210), “Outside of work, have you ever been exposed to firearms noise for an average of at least once a month for a year?” We note that this question is specific to firearm noise exposure. In the 2011–2012 and 2015–2016 survey cycles, we used the question (AUQ300), “This next question is about your use of firearms that you may have used for target shooting, hunting, for your job, or in military service. Have you ever used firearms for any reason?” to assess firearm use. We treated firearm noise exposure and firearm use as binary variables (yes/no). To characterize dose-response relations, we used a follow-up question (AUQ310), which was only available in 2011–2012 and 2015–2016, “How many total rounds have you ever fired? (1–100, 100–1000, 1000–10,000, 10,000–50,000, 50,000+)?” Due to the small sample size in the 10,000–50,000 and 50,000+ categories, we collapsed the number of rounds shot into 1–1,000, 1,000–10,000, and 10,000+.

### Lead exposure assessment

Our outcome was blood lead concentrations. In 1999–2004 and 2011–2012, venous blood samples were drawn and analyzed for lead from mobile examination participants above 1. In 2015–2016, venous blood samples were drawn from mobile examination participants above 1, but only half of the participants aged 12 and above had specimens analyzed for lead.

Blood lead specimens were stored and processed at the Division of Laboratory Sciences, National Center for Environmental Health, Centers for Disease Control and Prevention. Measurements were conducted using atomic absorption spectrometry (PerkinElmer Model SIMAA 6000, 1999–2002) or inductively coupled plasma dynamic reaction cell mass spectrometry (PerkinElmer ELAN 6100, 2003–2004; PerkinElmer ELAN DRC II, 2011–2012 and 2015–2016). Blood lead limits of detection (LODs) were 6 µg/L (1999–2002), 2.5 µg/L (2003–2004 and 2011–2012), and 0.7 µg/L (2015–2016).^33–37^ The National Center for Health Statistics replaced blood lead concentrations below the LOD with a value equal to the LOD/√2. For the 1999–2004 and 2011–2012, 2015–2016 survey cycles, 1.8% (n = 198) and 0.5% (n = 36) of blood lead concentrations were below the detection limit, respectively. For compatibility with other biomarkers, we converted all blood lead concentrations from µg/dL to µg/L (i.e., parts-per-billion).^38^

### Covariates

We selected gender, age, race/ethnicity, and education as covariates based on our Directed Acyclic Graph (DAG, Figure S1; https://links.lww.com/EE/A378). Participant age was continuous in years. Race/ethnicity was self-reported and followed US Census Bureau categorizations: Mexican American, other Hispanic, non-Hispanic White, non-Hispanic Black, and other race (1999–2004), with the addition of non-Hispanic Asian (2011-present). To avoid positivity violations, we collapsed race/ethnicity into Mexican American/other Hispanic, non-Hispanic White, non-Hispanic Black, and other race (non-Hispanic Asians included). NHANES categorized education as less than 9th grade, 9th–11th grade (includes 12th grade with no diploma), high school graduate/GED or equivalent, some college or AA (Associates of Arts) degree, and college graduate or above. For model parsimony, we collapsed education into less than high school (<HS), high-school graduate/General Education Degree or equivalent (HS/GED), and some college and above (college+).

Former military service, year the home was built (pre-1978, 1978-after), and occupation were included as covariates in separate sensitivity analyses. The year home was built was not included in the primary analyses because data were not available for 2011–2012 and 2015–2016. Occupation was not included because industry codes for 2011–2012 were inconsistent with 1999–2004, and industry-specific data were not available for 2015–2016.

### Statistical analyses

For all analyses, we followed NHANES analytic guidelines on complex survey design (https://wwwn.cdc.gov/nchs/nhanes/tutorials/weighting.aspx).^39^ For each survey cycle, we used the weight of the smallest included subpopulation (mobile examination participants or blood metal subsample participants). Because the 1999–2000 sample weights did not include population estimates from the 2000 US Census, we used 4-year mobile examination weights created by NHANES to account for sample weight differences between 1999–2000 and 2001–2002. For 1999–2004, we combined the 4-year mobile examination weights with 2-year mobile examination weights (⅔*WTMEC4YR for 1999–2002 and ⅓*WTMEC2YR for 2003–2004). Because the 2015–2016 survey cycle contained a subsample of participants selected for blood lead measurements, we combined 2-year mobile examination weights with 2-year blood metal subsample weights (½*WTMEC2YR for 2011–2012 and ½*WTSH2YR for 2015–2016). All analyses were conducted in R (version 2023.12.1+402, R Foundation, Vienna, Austria).^40^

We calculated univariate statistics of blood lead concentrations by survey cycles (1999–2004 and 2011–2012, 2015–2016). For the two sets of analyses, we also calculated median, 25th, and 75th percentile blood lead concentrations, and population-weighted percentages of participants exposed to firearms (1999–2004) or participants who used firearms (2011–2012 and 2015–2016) according to categories of covariates.

We used log_2_-transformed blood lead concentrations to approximate normality assumptions. Using multivariable linear regression, we examined the association between self-reported firearm noise exposure and log_2_-transformed blood lead concentrations in 1999–2004 before and after adjusting for covariates. For 2011–2012 and 2015–2016, we used multivariable linear regression to examine the association of both firearm use and number of rounds shot with log_2_-transformed blood lead concentrations. To assess the trend between number of rounds shot and log_2_-transformed blood lead concentrations, we conducted a linear trend test using a continuous variable with four possible values– 0 (no rounds shot), 500 (1–1,000 rounds), 5,000 (1,000–10,000 rounds), and 24,193 (10,000+ rounds; weighted average based on the proportion of participants who shot 10,000–50,000 rounds and 50,000 rounds+). We exponentiated the firearm variable beta coefficient to calculate percent difference in geometric mean blood lead concentrations in firearm-exposed or firearm-using participants compared to nonfirearm-exposed or nonfirearm-using participants. The nonfirearm-exposed and nonfirearm-using participants served as our referent group.

We conducted three separate sensitivity analyses to assess the potential for residual confounding. First, we excluded former military service members and adjusted for former military service. Former military service was categorized as service in the Armed Forces (1999–2004) or active-duty service in the Armed Forces, military reserves, or National Guard (2011–2012 and 2015–2016). Service includes part-time reservists, whereas active-duty service includes only full-time military members or reservists with at least 180 days of nontraining service.^41^ Second, after excluding participants without housing age data in 1999–2004, we adjusted for the year the participant’s home was built (pre-1978 or 1978-after). Third, after excluding participants without industry-specific occupation data in 1999–2004, we adjusted for occupation in military or public safety industries (categorized as “military & national security” or “justice, order, and public safety”).

We examined gender as a potential modifier given the observed gender-specific differences in self-reported firearm noise exposure and firearm use.^42^ To detect gender-specific effect measure modification, we estimated the *P* value from the production interaction term between gender and self-reported firearm noise exposure or firearm use.

## Results

Participants in 1999–2004 had higher median blood lead concentrations (15 µg/L; interquartile range [IQR] = 10–22) than participants in 2011–2012 and 2015–2016 (8.8 µg/L; IQR = 5.7–14) (Table S8; https://links.lww.com/EE/A378). In all time periods, males, those aged 50–69, and those with less than a high-school education had higher median blood lead concentrations than comparison groups in their respective categories (Table 1). Across race/ethnicity, median blood lead concentrations were similar in 1999–2004 (absolute differences ≤2 µg/L) (Table 1). In 2011–2012 and 2015–2016, blood lead concentrations were similar except for other race, which was highest (Table 1). The prevalence of self-reported firearm noise exposure (1999–2004) and self-reported firearm use (2011–2012 and 2015–2016) was greatest among males and non-Hispanic Whites (Table 1).

**Table 1.** Sample size, weighted median blood lead concentrations, and prevalence of self-reported firearm noise exposure (1999–2004) and firearm use (2011–2012 and 2015–2016) among US adults aged 20–69 by covariates

			Survey cycle			
		1999–2004			2011–2012, 2015–2016	
Covariate	n	Median blood lead µg/L (IQR)	Weighted % exposed to firearms	n	Median blood lead µg/L (IQR)	Weighted % using firearms
	9,606	15 (10, 22)	6.5	5,972	8.8 (5.7, 14)	44
Gender						
Male	3,948	18 (13, 27)	11	2,740	10 (6.9, 16)	59
Female	5,658	12 (8, 18)	3	3,232	7.8 (5.1, 12)	32
Age						
20–29	2,400	11 (7, 16)	6.7	1,263	5.9 (4.3, 9.3)	44
30–39	2,128	14 (9, 20)	7.1	1,284	7.0 (4.7, 11)	46
40–49	2,042	16 (11, 23)	6.6	1,195	8.8 (6.2, 14)	43
50–59	1,404	18 (13, 26)	5.9	1,139	12 (8.4, 18)	46
60–69	1,632	20 (13, 28)	5.0	1,091	13 (8.8, 19)	40
Race/ethnicity						
Non-Hispanic Black	1,919	16 (11, 25)	3.4	1,441	8.7 (5.7, 15)	20
Non-Hispanic White	4,288	14 (9, 21)	7.4	1,884	8.7 (5.6, 13)	56
Other race	387	15 (10, 24)	7.1	1,083	11 (7.0, 16)	28
Mexican/other Hispanic	3,012	16 (10, 25)	4.1	1,564	8.3 (5.4, 13)	23
Education level						
<HS	3,048	19 (12, 29)	6.0	1,343	11 (7.1, 18)	29
HS/GED	2,222	15 (10, 23)	8.5	1,236	9.1 (5.6, 16)	42
Some college+	4,336	13 (9, 20)	5.7	3,393	8.3 (5.5, 13)	48

GED indicates General Education Development certification; HS, high school.

In the 1999–2004 data examined before adjustment for covariates, self-reported firearm noise exposure was associated with a 30% increase in blood lead concentrations (95% CI = 20%, 41%) compared to nonfirearm-noise-exposed groups (Table 2). Though attenuated, self-reported firearm noise exposure was still associated with a modest increase in blood lead concentrations (15%; 95% CI = 7%, 23%) (Table 2) after adjustment for gender, age, race/ethnicity, and education.

**Table 2.** Unadjusted and adjusted percent differences in blood lead concentration (µg/L) by self-reported firearm noise exposure status, 1999–2004^a^

Firearm exposure	n (weighted %)	Unadjusted GM blood lead conc.	Unadjusted % difference, (CI)	Adjusted % difference, (CI)	*P* value^b^
No	9098 (93)	14	--	--	--
Yes	508 (7)	19	30% (20%, 41%)	15% (7%, 23%)	0.0003

a Adjusted for gender (male or female), age (continuous), race/ethnicity (non-Hispanic Black, non-Hispanic White, other race, Mexican American/other Hispanic), and education (<HS, HS/GED, > some college).

b *P* value for adjusted model.

GM indicates geometric mean.

**Figure 1.**
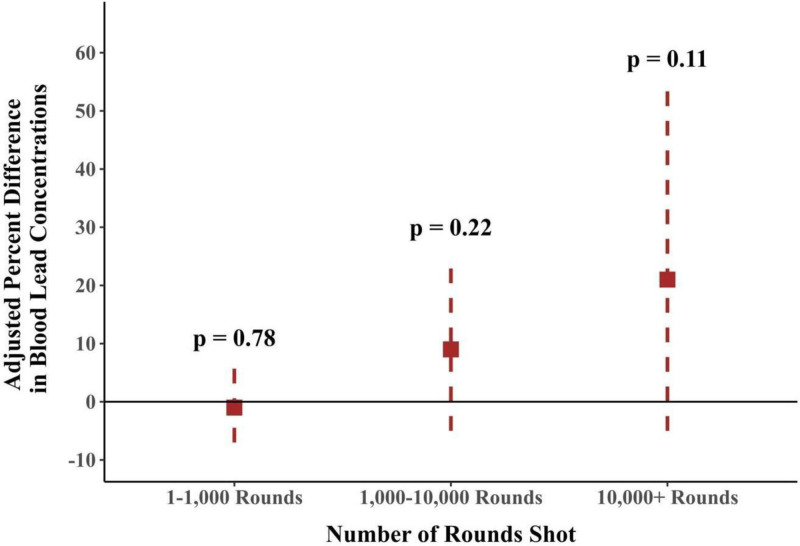
Forest plot of adjusted percent differences in blood lead concentrations (95% CI) based on number of rounds shot: National Health and Nutrition Examination Survey 2011–2012 and 2015–2016^a,b,c a^Adjusted for gender (male or female), age (continuous), race/ethnicity (non-Hispanic Black, non-Hispanic White, other race, Mexican American/other Hispanic), and education (<HS, HS/GED, > some college). ^b^Number of rounds shot represented as median number of rounds shot per category (1–1,000 rounds and 1,000–10,000 rounds) and weighted average (10,000+ rounds). ^c^*P* = 0.07 for adjusted linear trend test.

Similar to findings from 1999 to 2004 (Table 2), adjustment for covariates attenuated the association between firearm use and blood lead concentrations in the 2011–2012 and 2015–2016 cycles (Table S1; https://links.lww.com/EE/A378), but firearm use was no longer associated with blood lead concentrations after adjustment (1%; 95% CI = −5%, 9%) (Tables S1; https://links.lww.com/EE/A378). However, we observed evidence that those who report shooting more rounds had higher blood lead concentrations compared with those who report shooting zero rounds (1–1000, −1%; 1,000–10,000, 9%; 10,000+, 21%; *P* value for trend = 0.07) (Figure 1 and Table S1; https://links.lww.com/EE/A378).

Results were similar after adjusting for former military service, year home was built (pre-1978 or 1978-after), and occupation in military or public safety industries (Tables S2–S5; https://links.lww.com/EE/A378). Inclusion of former military service members in 2011–2012 and 2015–2016 did not change the dose-response relationship between firearm use and blood lead concentrations (*P* value for trend = 0.08) (Table S3; https://links.lww.com/EE/A378).

The associations of self-reported firearm noise exposure and firearm use with blood lead concentrations were similar in gender-stratified analyses compared with primary findings (Tables S6–S7; https://links.lww.com/EE/A378).

## Discussion

Among noninstitutionalized US civilians aged 20–69, self-reported firearm noise exposure (a proxy for firearm exposure) was associated with elevated blood lead concentrations. When examining self-reported firearm use, elevated blood lead concentrations were only observed among those reporting using 10,000+ rounds compared with those using less. Additional adjustments for former military service, year home was built, and occupation did not substantially change our findings.

Blood lead concentrations have decreased among US civilians over the last two decades because of declines in legacy-based lead exposure sources.^12,43,44^ Leaded paint, leaded gasoline, and lead water lines were phased out by the US Environmental Protection Agency in the late 1970s and early 1980s.^12,43,44^ Thus, geometric mean blood lead concentrations of US adults decreased from 16.8 µg/L to 8.2 µg/L between 1999 and 2016, a trend similarly observed in our sample.^45^ However, within certain groups and industries, lead exposure still remains prevalent at levels associated with adverse health effects.^43^ Due to declines in legacy-based lead exposure sources, contemporary sources of lead, such as those related to firearms, may represent an increased proportion of population-level lead exposure.^46^ An estimated 15% of the US adult population participates regularly in target shooting, and except for states like California, which banned lead ammunition for hunters in 2019, lead ammunition is a major source of unregulated lead in the US environment.^16,43,47–50,46^ As such, our study, which analyzes firearm-related lead exposure among noninstitutionalized US citizens, presents novel insight into this potentially modifiable source of lead exposure, which has primarily been investigated in military, police, or other noncivilian settings.

Our findings are consistent with studies that describe an association of firearm exposure or use with blood lead concentrations.^24,51,52^ In an Italian study with 376 firearm-exposed participants (firearm instructors) and 170 nonfirearm-exposed participants (food plant workers), 23% of firearm-exposed participants had blood lead concentrations above the 95th percentile of the Italian adult reference population (100 µg/L), compared to only 0.6% of nonfirearm-exposed participants.^24^ Prior studies, including a cross-sectional study of 78 Swedish police officers, also found a dose-response relationship between levels of firearm use and blood lead concentrations.^25,53^ In comparison to officers who fired 999 rounds annually, officers who fired 1,000–2,999, 3,000–9,999, and 10,000+ rounds annually had blood lead concentrations 8.3, 16.6, and 22.8 µg/L greater, respectively.^25^ In a South African study that compared 87 recreational firearm users to 31 nonfirearm users (archers), firearm users were more likely to have blood lead concentrations >100 µg/L.^17^ Other studies assessed changes in blood lead concentrations before and after firearm training courses, in which blood lead concentrations increased significantly compared to baseline.^23,29–31,54^

One reason our findings may not have established as consistent of an association between firearm use and blood lead concentrations is that past studies primarily focused on occupational exposures, particularly in military or police settings.^23,26–31,55,56^ Military or police firing ranges may have more controlled operations, protective measures, and weaponry that differ from recreational firing or hunting ranges.^57,58^ Military service members and police personnel also interact with and use firearms in different contexts compared with civilians.^56,59^ Thus, most current literature on firearm use and blood lead concentrations may not be generalizable to the noninstitutionalized civilian population. Furthermore, studies with a majority of male participants do not fully reflect the noninstitutionalized civilian population.^9,17,23–27,29,30,53,60,61^

Our study has limitations. First, the use of self-reported firearm noise exposure as a proxy for firearm-related lead exposure is not a direct measure of firearm use. However, we believe this is a valid proxy, as the prevalence of firearm noise exposure by covariates in our study (i.e., greatest for male and non-Hispanic White participants) mirrors US firearm owner demographics and was similar to the patterns observed for the more specific question about firearm use administered in the 2011–2012 and 2015–2016 NHANES.^62^ Furthermore, firearm noise exposure may capture additional sources of individual-level exposure beyond firearm use. Our measure required monthly exposure to firearms over a continuous 12-month period, reflecting repeated exposure. In contrast, our measure of firearm use is binary and categorizes participants with any use of firearms, regardless of frequency or duration, as users. This difference in wording may explain why we observed a stronger association between self-reported firearm noise exposure and blood lead concentrations than between firearm use and blood lead concentrations. Relatedly, this may explain why we observed associations only among those self-reporting using 10,000+ rounds, as they would be more consistent firearm users and thus have chronic exposure.

Second, our study is cross-sectional, which limits causal inference. Third, the limited sample size in the 10,000+ rounds category was small and thus reduced statistical precision. Fourth, participants self-reported all firearm-related questionnaire data, which may induce recall bias. Moreover, identification of firearm noise itself may be subjective, based on participants’ sensitivity to noise, general location, or other ambient noise levels.^63^ Participants may misremember firearm noise exposure frequencies, potentially overestimating more recent exposures and underestimating more distant ones. Furthermore, participants who use firearms more frequently may better recall their use behaviors compared with participants who do not use firearms as frequently. This bias may therefore induce some degree of differential misclassification. The NHANES did not measure participants’ actual proximity to firearms and subsequent lead exposure, so we cannot determine whether possible exposure misclassification was random or related to an unmeasured confounder. However, given the adjustments we made for key predictors of lead exposure (e.g., sex, age, race/ethnicity, education, former military service, year home was built, occupation), we attempted to mitigate the extent to which confounding might bias our results. Fifth, our measure of lead exposure (blood lead concentration) only reflects recent (30–35 day) exposures and not chronic exposures, which may be more predictive of the long-term health effects of lead.^64^

Since we replicated analyses across multiple survey cycles, additional limitations include minor differences in question wording between cycles. While the 1999–2004 survey cycles assessed nonoccupational firearm noise exposure, the 2011–2012 and 2015–2016 survey cycles considered occupational firearm use. While the 1999–2004 survey cycles defined military service as any service in the United States Armed Forces, the 2011–2012 and 2015–2016 survey cycles defined military service as active-duty service in the US Armed Forces, military reserves, or National Guard.^41^ However, inclusion of former military service members in separate sensitivity analyses did not change findings. Availability of data was not always consistent between survey cycles (e.g., the year a participant’s home was built was not available for 2011–2012 and 2015–2016). However, results from a housing-based sensitivity analysis for 1999–2004 mirrored primary findings. Similarly, though coded occupation data was not available for the 2011–2012 and 2015–2016 survey cycles, occupation-based sensitivity analyses for 1999–2004 did not change findings.

Despite these limitations and the relatively modest associations observed in our study, prior research suggests that any amount of lead is associated with adverse health outcomes, even at levels within an order of magnitude observed in our study. For example, decreased memory and cognitive function, increased odds of major depressive disorder, decreased renal function, increased cardiovascular disease, adverse pregnancy outcomes, and more.^5,13,65,66–75^ In a cross-sectional analysis of the 1999–2002 NHANES, adults with blood lead concentrations above 24 µg/L had a higher odds of having chronic kidney disease (OR = 2.72; 95% CI = 1.47, 5.04) and peripheral arterial disease (OR = 1.92; 95% CI = 1.02, 3.61) compared with those with blood lead concentrations below 10 µg/L.^73^ Thus, increases in blood lead concentrations associated with firearm exposure and use could adversely affect the health and well-being of US adults.

## Conclusions

Despite the limitations, our study is among the first to examine the association of self-reported firearm noise exposure, a proxy for firearm-related lead exposure, and firearm use with blood lead concentrations in a nationally representative sample of US civilians aged 20–69. These results suggest that frequent firearm use may increase lead exposure in civilians. Future studies should confirm our findings, particularly with respect to duration and frequency of use, and identify factors that modify firearm-related lead exposure.

## Conflicts of interest statement

J.B. was compensated for serving as an expert witness on behalf of PFAS-related drinking water litigation. The other authors declare that they have no conflicts of interest with regard to the content of this report.

## Acknowledgements

We would like to acknowledge the support of Karl T. Kelsey, MD, MOH, for providing valuable feedback on the final draft of this manuscript.

## Supplementary Material


