# Supplementary material for: Cross-sectional associations of self-reported firearm use with blood lead concentrations in a nationally representative cohort of US adults
Source: Environ Epidemiol. 2025 Oct 23;9(6):e427. doi: 10.1097/EE9.0000000000000427 (PMC12551738; doi:10.1097/EE9.0000000000000427)
Supplement: Supplementary file 1 [file ee9-9-e427-s001.pdf]

Supplemental Material:

Cross-Sectional Associations of Self-Reported Firearm Use with Blood Lead Concentrations in a Nationally Representative Cohort of US Adults

Authors: Madeline Day,[a] Joseph M. Braun,[a] RN, MSPH, PhD, Christian Hoover,[a,b] MPH

[a]Brown University, School of Public Health, Department of Epidemiology, 121 S. Main St, Providence, RI 02903

[b]Harvard University Injury Control Research Center, Harvard T.H. Chan School of Public Health, 677 Huntington Ave, Boston, MA 02115

Figures: 1

Tables: 8

Figure S1: Directed acyclic graph (DAG) depicting causal pathway between firearm use and blood lead concentrations[a,b,c]

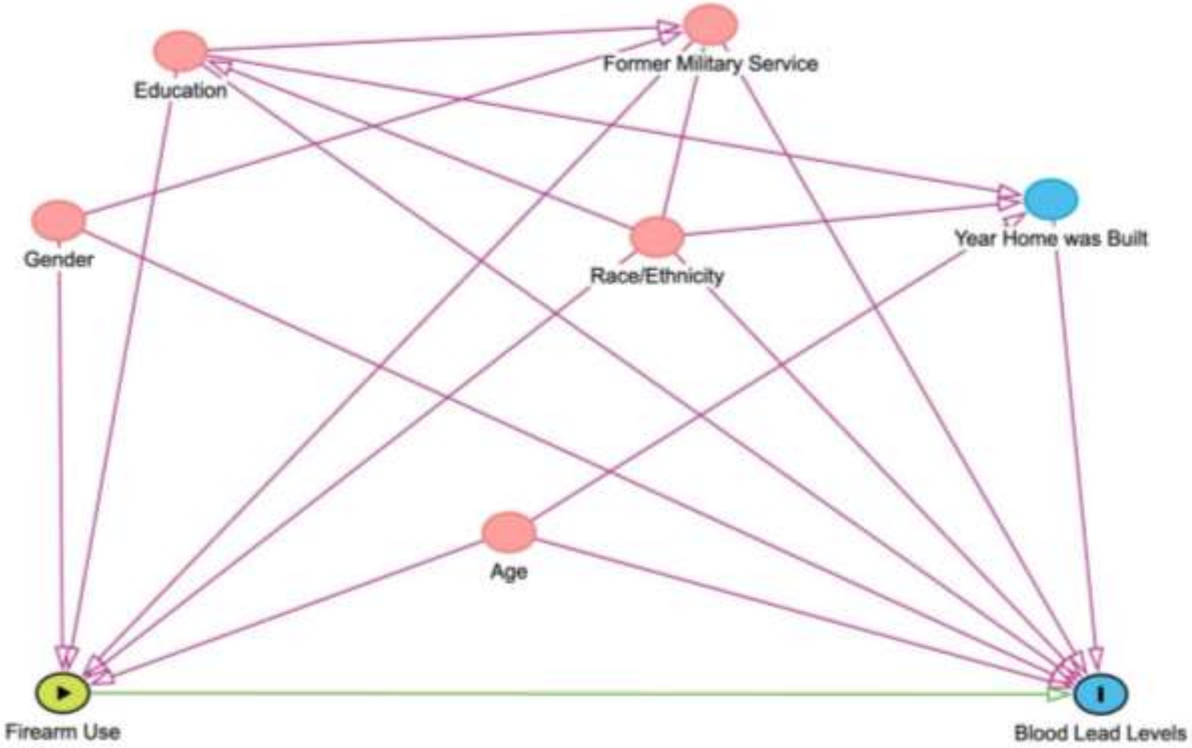

[a]Green = exposure, blue (I) = outcome, blue = ancestor of outcome, pink = ancestor of exposure and outcome
[b]Race/ethnicity refers to sociodemographic and structural determinants, not biological attributes
[c]Former military service and year home was built are covariates in separate sensitivity analyses

Table S1: Unadjusted and adjusted percent differences in blood lead concentration (ug/L) by number of rounds shot, 2011-2012 and 2015-2016[a]

Number of Rounds Shot[b]	n (weighted %)	Unadjusted GM[e] Blood Lead Conc., ug/L	Unadjusted % Difference, (CI)	Adjusted % Difference, (CI)	p-value[c]
0	4,073 (56)	9.0	--	--	--
1-1000	1,492 (34)	8.9	-1% (-7%, 5%)	-1% (-7%, 6%)	0.78
1000-10,000	283 (7)	10.6	17% (6%, 28%)	9% (-5%, 25%)	0.22
10,000+	124 (3)	13.6	50% (20%, 87%)	21% (-5%, 54%)	0.11
Yes	1,899 (44)	9.4	4% (-1%, 10%)	1% (-5%, 9%)	0.68[d]

[a]Adjusted for gender (male or female), age (continuous), race/ethnicity (non-Hispanic Black, non-Hispanic White, Other race, Mexican American/Other Hispanic), and education (<HS, HS/GED, > some college).
[b]Self-reported within participants' lifetime
[c]p-value for adjusted model
[d]p = 0.07 for adjusted linear trend test
[e]GM = geometric mean

Table S2: Unadjusted and adjusted percent differences in blood lead concentration (ug/L) by self-reported firearm noise exposure status (former military service[a] members included), 1999-2004[b]

Firearm exposure	n (weighted %)	Unadjusted GM[d] Blood Lead Conc.,	Unadjusted % Difference, (CI)	Adjusted % Difference, (CI)	p-value[c]
No	10,050 (91)	15	--	--	--
Yes	773 (9)	20	30% (22%, 38%)	11% (5%, 17%)	0.0004

[a]Service in the US Armed Forces, n =1,217

[b]Adjusted for gender (male or female), age (continuous), race/ethnicity (non-Hispanic Black, non-Hispanic White, Other race, Mexican American/Other Hispanic), education (<HS, HS/GED, > some college), and former military service (yes/no)

[c]p-value for adjusted model

[d]GM = geometric mean

Table S3: Unadjusted and adjusted percent differences in blood lead concentration (ug/L) by number of rounds shot (former military service[a] members included), 2011-2012 and 2015-2016[b]

Number of Rounds Shot[c]	n (weighted %)	Unadjusted GM[f] Blood Lead Conc., ug/L	Unadjusted % Difference, (CI)	Adjusted % Difference, (CI)	p-value[d]
0	4,100 (52)	9.1	--	--	--
1-1000	1,654 (34)	9.1	0% (-6%, 6%)	-1% (-8%, 6%)	0.71
1000-10,000	411 (9)	11	19% (9%, 30%)	6% (-6%, 19%)	0.33
10,000+	242 (5)	13	37% (17%, 61%)	16% (-4%, 39%)	0.11
Yes	2,307 (48)	9.7	7% (1%, 12%)	0% (-6%, 8%)	0.86 [d]

[a]Active-duty service in the US Armed Forces, military reserves, or National Guard, n = 435
[b]Adjusted for gender (male or female), age (continuous), race/ethnicity (non-Hispanic Black, non-Hispanic White, Other race, Mexican American/Other Hispanic), education (<HS, HS/GED, > some college), and former military service (yes/no)
[c]Self-reported within participants' lifetime
[d]p-value for adjusted model
[e]p = 0.08 for adjusted linear trend test
[f]GM = geometric mean

Table S4: Unadjusted and adjusted percent differences in blood lead concentration (ug/L) by self-reported firearm noise exposure status (adjusted for year home was built[a]), 1999-2004[b]

Firearm exposure	n (weighted %)	Unadjusted GM[d] Blood Lead Conc.,	Unadjusted % Difference, (CI)	Adjusted % Difference, (CI)	p-value[c]
No	6,760 (94)	14	--	--	--
Yes	391 (6)	19	30% (20%, 41%)	13% (6%, 21%)	0.0008

[a]n = 2,455 excluded due to lack of housing data

[b]Adjusted for gender (male or female), age (continuous), race/ethnicity (non-Hispanic Black, non-Hispanic White, Other race, Mexican American/Other Hispanic), education (<HS, HS/GED, > some college), and year home was built (pre-1978, 1978-after).

[c]p-value for adjusted model

[d]GM = geometric mean

Table S5: Unadjusted and adjusted percent differences in blood lead concentration (ug/L) by self-reported firearm noise exposure status (adjusted for occupation in military or public service industries[a]), 1999-2004[b]

Firearm exposure	n (weighted %)	Unadjusted GM[d] Blood Lead Conc.,	Unadjusted % Difference, (CI)	Adjusted % Difference, (CI)	p-value[c]
No	6,212 (98)	14	--	--	--
Yes	95 (2)	19	34% (22%, 47%)	15% (6%, 25%)	0.001**

[a]Justice, public order, and safety, or military and national security industries, n = 95
[b]Adjusted for gender (male or female), age (continuous), race/ethnicity (non-Hispanic Black, non-Hispanic White, Other race, Mexican American/Other Hispanic), education (<HS, HS/GED, > some college), and occupation (military or public service industries, all other industries)
[c]p-value for adjusted model
[d]GM = geometric mean

Table S6: Gender-stratified unadjusted and adjusted percent differences in blood lead concentration (ug/L) by self-reported firearm noise exposure status by sex, 1999-2004[a]

Firearm exposure	n (weighted %)	Unadjusted GM[c] Blood Lead Conc.,	Unadjusted % Difference, (CI)	Adjusted % Difference, (CI)	p-value[b]
**Males**					
No	3591 (89)	19	--	--	--
Yes	357 (11)	22	16% (6%, 26%)	16% (7%, 26%)	0.001**
**Females**					
No	5507 (97%)	12.4	--	--	--
Yes	151 (3%)	13.3	8% (-3%, 20%)	10% (-1%, 23%)	0.07

[a]Adjusted for age (continuous), race/ethnicity (non-Hispanic Black, non-Hispanic White, Other race, Mexican American/Other Hispanic), and education (<HS, HS/GED, > some college)
[b]p-value for adjusted model
[c]GM = geometric mean
[d]Effect-measure modification p-value: 0.29

Table S7: Gender-stratified unadjusted and adjusted percent differences in blood lead concentration (ug/L) by number of rounds shot, 2011-2012 and 2015-2016[a]

Number of Rounds Shot[b]	n (weighted %)	Unadjusted GM[g] Blood Lead Conc., ug/L	Unadjusted % Difference, (CI)	Adjusted % Difference, (CI)	p-value
**Males**					
0	1482 (41)	11.0	--	--	--
1-1000	897 (39)	10.2	-7% (-16%, 3%)	0% (-11%, 12%)	0.96
1000-10,000	244 (14)	11.1	1% (-12%, 17%)	11% (-7%, 33%)	0.23
10,000+	117(6)	13.7	25% (-1%, 57%)	22% (-5%, 57%)	0.12[d]
Yes	1258 (59)	10.7	-2% (-12%, 8%)	4% (-8%, 18%)	0.51[c]
**Females**					
0	2591 (68)	8.2	--	--	--
1-1000	595 (29)	7.7	-6% (-15%, 3%)	-1% (-9%, 7%)	0.79
1000-10,000	39 (2)	7.7	-6% (-18%%, 9%)	5% (-5%, 17%)	0.32
10,000+	7 (0.4)	11.7	42% (-5%, 113%)	17% (-21%, 73%)	0.42[e]
Yes	641 (32)	7.7	-6% (-14%, 3%)	0% (-8%, 7%)	0.90[f]

[a]Adjusted for age (continuous), race/ethnicity (non-Hispanic Black, non-Hispanic White, Other race, Mexican American/Other Hispanic), and education (<HS, HS/GED, > some college).
[b]Self-reported within participants' lifetime
[c,f]p-value for adjusted model
[d]p-value for adjusted linear trend test = 0.08
[e]p-value for adjusted linear trend test = 0.31
[g]GM = geometric mean

Table S8: Univariate characteristics of blood lead concentrations (ug/L) among participants[a] based on survey cycles, 1999-2004 and 2011-2012, 2015-2016

Survey Cycles	Min	25th percentile	Median	75th percentile	Max
1999-2004	2	10	15	22	540
2011-2012, 2015-2016	0.5	5.7	8.8	14	613

[a]Participants from primary analyses only
